# 

**Published:** 1886-05

**Authors:** 


### American Public Health Association.

The next (14th) annual meeting of this Association will be held in Toronto, Ont., October 6–8, 1886.

The Executive Committee have selected the following topics for consideration at said meeting:

I. The Disposal of the Refuse Matters of Cities and Towns.

II. The Condition of Stored Water-Supplies, and their Relation to the Public Health.

III. The Best Methods and the Apparatus Necessary for the Teaching of Hygiene in the Public Schools, as well as the Means for Securing Uniformity in such Instruction.

IV. Recent Sanitary Experiences in connection with the Exclusion and Suppression of Epidemic Disease.

V. The Sanitary Conditions and Necessities of School-Houses and School-Life. (See Lomb Prize Essays.)

VI. The Preventable Causes of Disease, Injury, and Death in American Manufactories and Workshops, and the Best Means and Appliances for Preventing and Avoiding them. (See Lomb Prize Essays.)

VII. Plans for Dwelling-Houses. (See Lomb Prize Essays.)

### Notice of Removal.

Dr. T. Gaillard Thomas has removed from 294 Fifth avenue, New York, to 600 Madison avenue, between Fifty-seventh and Fifty-eighth streets.

